# The TIP60-ATM axis regulates replication fork stability in BRCA-deficient cells

**DOI:** 10.1038/s41389-022-00410-w

**Published:** 2022-06-18

**Authors:** Emily M. Schleicher, Ashna Dhoonmoon, Lindsey M. Jackson, Jude B. Khatib, Claudia M. Nicolae, George-Lucian Moldovan

**Affiliations:** Department of Biochemistry and Molecular Biology, The Pennsylvania State University College of Medicine, Hershey, PA 17033 USA

**Keywords:** DNA damage and repair, Genomic instability

## Abstract

Maintenance of replication fork stability is essential for genome preservation. Stalled replication forks can be reversed by translocases such as SMARCAL1, and unless protected through the activity of the BRCA pathway, are subsequently subjected to nucleolytic degradation. The ATM and ATR kinases are master regulators of the DNA damage response. ATM activation upon DNA damage is mediated by the acetyltransferase TIP60. Here, we show that the TIP60-ATM pathway promotes replication fork reversal by recruiting SMARCAL1 to stalled forks. This enables fork degradation in BRCA-deficient cells. We also show that this ATM activity is not shared by ATR. Moreover, we performed a series of genome-wide CRISPR knockout genetic screens to identify genetic determinants of the cellular sensitivity to ATM inhibition in wildtype and BRCA2-knockout cells, and validated the top hits from multiple screens. We provide a valuable list of common genes which regulate the response to multiple ATM inhibitors. Importantly, we identify a differential response of wildtype and BRCA2-deficient cells to these inhibitors. In BRCA2-knockout cells, DNA repair genes (including RAD17, MDC1, and USP28) were essential for survival upon ATM inhibitor treatment, which was not the case in wild-type cells. These findings may eventually help guide the way for rational deployment of ATM inhibitors in the clinic.

## Introduction

During DNA replication, obstacles that block the progression of DNA polymerases pose a major risk as they arrest the replication fork. Arrested forks can be nucleolytically processed into double-stranded breaks resulting in genomic instability [1, 2]. To avoid this, stalled forks can be processed in a number of different ways to promote their stabilization and restart. In recent years, reversal of stalled replication forks by annealing the nascent strands of the two sister chromatids to each other, has emerged as an important fork processing event [3–10]. Fork reversal is catalyzed by DNA translocases including HLTF, ZRANB3 and SMARCAL1, and allows the opportunity for DNA replication to restart using the nascent strand of the sister chromatid as the template.

At the same time, reversed replication forks are subjected to nucleolytic processing by MRE11 and other nucleases, which can also lead to genomic instability [5–7, 9, 11, 12]. The activity of the BRCA tumor suppressor pathway is essential for protection of reversed forks against nucleolytic degradation, through a process which, at least in part, is thought to involve the loading of RAD51 onto reversed forks thereby blocking nuclease engagement [11, 12]. Conversely, degradation of stalled replication forks is a hallmark of BRCA deficiency, and may contribute to the genomic instability observed in these cells; Moreover, it may also contribute to their hypersensitivity to genotoxic agents, possibly including those employed in cancer therapy such as cisplatin and PARP inhibitors (PARPi) [6, 11–13].

Genome-wide CRISPR genetic screens have recently emerged as a powerful tool to identify biomarkers of the cancer cells’ response to chemotherapeutic agents. Moreover, CRISPR screens designed to identify genetic determinants of the cellular response to inhibitors of DNA repair enzymes can unveil important insights into DNA repair mechanisms and regulation [14]. We recently identified the acetyltransferase TIP60 (also known as KAT5) as a regulator of chemoresistance in BRCA2-deficient cells [15]. Loss of TIP60 suppresses cisplatin and PARPi hypersensitivity of these cells, and promotes 53BP1-mediated non-homologous end joining (NHEJ) repair of double-stranded DNA breaks (DBSs) [15–17].

ATM and ATR are two checkpoint kinases which regulate the cellular response to genotoxic stress [18–22]. ATM has been traditionally studied in the context of double-strand breaks, and TIP60 was shown to be specifically required for ATM activation, by catalyzing an activatory acetylation of ATM at Lys3016 [23, 24]. In contrast, ATR responds to replication-associated stress. However, the two kinases phosphorylate similar substrate motifs and share a large number of substrates [25]. Specific inhibitors of ATM and ATR have been developed and are making their way into the clinic. Several ATM inhibitors (ATMi) including AZD1390, AZD0156, and KU60019 are currently in early-phase clinical trials for treatment of solid tumors [26, 27]. For example, AZD1390 is in a phase I trial in combination with radiation therapy for treatment of brain tumors, while AZD0156 is in a phase I trial as a monotherapy or in combination with cytotoxic chemotherapies such as the PARP inhibitor (PARPi) olaparib in advanced cancers. Moreover, significant progress has been made in pre-clinical studies to identify the best application of these inhibitors. For instance, it was shown that pharmacological inhibition of ATM sensitized pancreatic dual adenocarcinoma to ATR inhibitors (ATRi) and gemcitabine [28]. Additionally, ATM inhibition was found to impede repair of olaparib-induced DNA damage, resulting in cell death in a panel of lung, gastric, and breast cancer cell lines [29].

Here, we show that the TIP60-ATM axis is essential for fork reversal, by promoting the recruitment of the SMARCAL1 translocase to stalled replication forks. Genetic or pharmacological inactivation of ATM suppresses fork reversal, and protects against fork degradation in BRCA-deficient cells. Through a series of genome-wide CRISPR knockout genetic screens, we moreover show that BRCA2-deficient cells have a different set of genetic determinants of ATMi sensitivity compared to wild-type cells, particularly relying on DNA repair for survival. This differential response to ATM inhibition may potentially prove relevant for treatment strategies of BRCA-mutant tumors.

## Results

### The TIP60-ATM pathway promotes replication fork reversal and degradation in BRCA-deficient cells

**Fig. 1:**
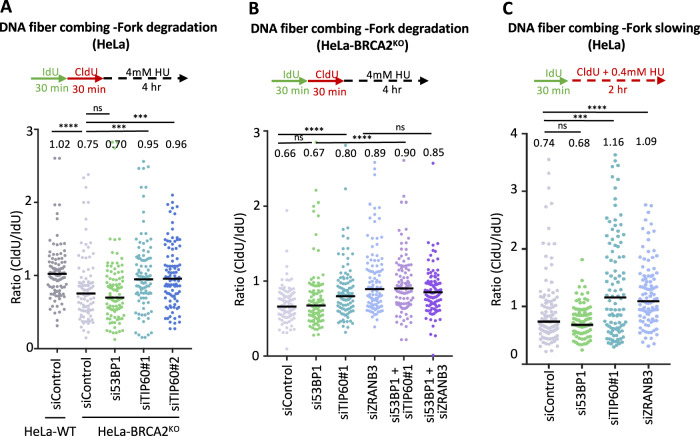
Loss of TIP60 suppresses stalled fork degradation in BRCA2-knockout cells in a 53BP1-independent manner. **A** DNA fiber combing assays showing that TIP60 depletion suppresses HU-induced fork degradation in BRCA2-knockout HeLa cells. The ratio of CldU to IdU tract lengths is presented, with the median values marked on the graph and listed at the top. At least 100 tracts were quantified for each sample. Asterisks indicate statistical significance (Mann–Whitney test). A schematic representation of the assay conditions is shown at the top. Western blots confirming the knockdown are shown in Supplementary Fig. S1A. **B** DNA fiber combing assays showing that 53BP1 is not required for fork protection mediated by TIP60 loss in BRCA2-deficient cells. 53BP1 depletion in HeLa-BRCA2^KO^ does not affect fork degradation, and does not affect the suppression of fork degradation induced by TIP60 knockdown in these cells. Depletion of the translocase ZRANB3 which catalyzes fork reversal is used as a control, since only reversed forks are subjected to degradation. The ratio of CldU to IdU tract lengths is presented, with the median values marked on the graph and listed at the top. At least 100 tracts were quantified for each sample. Asterisks indicate statistical significance (Mann–Whitney test). A schematic representation of the assay conditions is shown at the top. Western blots confirming the knockdown are shown in Supplementary Fig. S1B. **C** DNA fiber combing assay showing that TIP60 depletion in HeLa cells impairs HU-induced fork slowing. Knockdown of ZRANB3, which suppresses fork reversal, is shown as control. The ratio of CldU to IdU tract lengths is presented, with the median values marked on the graph and listed at the top. At least 100 tracts were quantified for each sample. Asterisks indicate statistical significance (Mann–Whitney test). A schematic representation of the assay conditions is shown at the top.

We previously identified TIP60 as a top hit in a genome-wide CRISPR knockout screen designed to uncover genetic determinants of chemoresistance in BRCA-deficient cells [15]. We found that loss of TIP60 results in resistance to the PARP inhibitor olaparib, as well as to the genotoxic chemotherapeutic agent cisplatin. Mechanistically, we showed that loss of TIP60 enhances 53BP1 binding to DSBs induced by these agents, promoting their repair through NHEJ, thus explaining the resistance to these agents conferred by TIP60 inactivation. In addition, we also found that loss of TIP60 suppresses nascent DNA degradation, which is a hallmark of BRCA deficiency [11, 12]. Protection of stalled forks against nucleolytic degradation has previously been associated with chemoresistance in BRCA-deficient cells [13]. However, to what extent fork protection conferred by TIP60 loss participates to chemoresistance under these conditions, remained unclear. To address this, we sought to investigate the impact of 53BP1 on fork protection, since we previously showed that the presence of 53BP1 was required for chemoresistance induced by TIP60 loss [15]. In line with our previous findings [15], DNA fiber combing experiments showed that loss of TIP60 suppressed hydroxyurea (HU)-induced fork degradation in BRCA2-knockout cells; In contrast, 53BP1 depletion did not have any impact on this degradation (Fig. 1A; Supplementary Fig. S1A). Importantly, co-depletion of 53BP1 did not restore fork degradation in TIP60-knockdown HeLa-BRCA2^KO^ cells (Fig. 1B; Supplementary Fig. S1B). This indicates that, unlike its activity in chemoprotection we previously documented [15], the role of TIP60 in fork protection does not involve its inhibition of 53BP1 binding to damaged DNA.

A prerequisite for fork degradation is the reversal of stalled replication forks through the annealing of the two nascent strands catalyzed by DNA translocases SMARCAL1, HLTF, and ZRANB3, creating the 4-way junction DNA structure which is the subject of degradation by nucleases MRE11, DNA2 and others [5–7, 9, 11, 12]. In line with these previous findings, ZRANB3 depletion suppressed fork degradation in HeLa-BRCA2^KO^ cells (Fig. 1B; Supplementary Fig. S1B). We thus sought to investigate if TIP60 loss operates in fork protection through a similar mechanism, namely by suppressing fork reversal. To address this, we employed the DNA fiber combing assay to measure the slowing of replication forks in the presence of low-dose HU, which was previously employed as a surrogate readout for fork reversal [7]. Similar to loss of ZRANB3, TIP60 inactivation suppressed HU-induced fork slowing in HeLa cells (Fig. 1C), suggesting a role for TIP60 in promoting fork reversal.

**Fig. 2:**
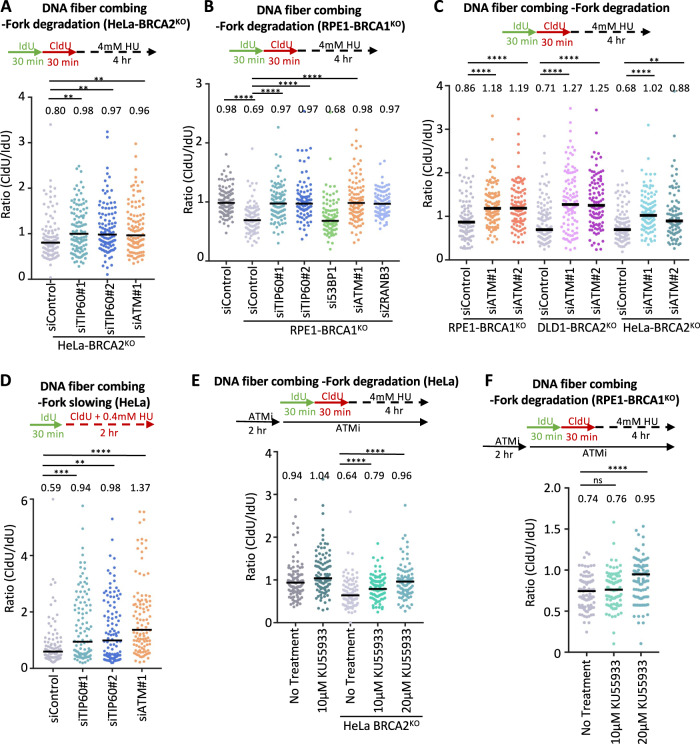
ATM inactivation inhibits fork reversal and protects against fork degradation in BRCA-deficient cells. **A**, **B** DNA fiber combing assays showing that, similar to TIP60 knockdown, ATM depletion suppresses HU-induced fork degradation in HeLa-BRCA2^KO^ (**A**) and RPE1-BRCA1^KO^ (**B**) cells. The ratio of CldU to IdU tract lengths is presented, with the median values marked on the graph and listed at the top. At least 100 tracts were quantified for each sample. Asterisks indicate statistical significance (Mann–Whitney test). A schematic representation of the assay conditions is shown at the top. Western blots confirming the knockdown are shown in Supplementary Fig. S1C. **C** DNA fiber combing assays showing that ATM depletion with two different siRNA oligonucleotides suppresses HU-induced fork degradation in HeLa-BRCA2^KO^, DLD1-BRCA2^KO^ cells, and RPE1-BRCA1^KO^ cells. The ratio of CldU to IdU tract lengths is presented, with the median values marked on the graph and listed at the top. At least 100 tracts were quantified for each sample. Asterisks indicate statistical significance (Mann–Whitney test). A schematic representation of the assay conditions is shown at the top. **D** DNA fiber combing assay showing that, similar to TIP60 depletion, ATM knockdown impairs HU-induced fork slowing in HeLa cells. The ratio of CldU to IdU tract lengths is presented, with the median values marked on the graph and listed at the top. At least 100 tracts were quantified for each sample. Asterisks indicate statistical significance (Mann–Whitney test). A schematic representation of the assay conditions is shown at the top. **E**, **F** DNA fiber combing assays showing that, similar to ATM knockdown, its pharmacological inhibition using the specific inhibitor KU55933 suppresses HU-induced fork degradation in HeLa-BRCA2^KO^ (**E**) and RPE1-BRCA1^KO^ (**F**) cells. The ratio of CldU to IdU tract lengths is presented, with the median values marked on the graph and listed at the top. At least 100 tracts were quantified for each sample. Asterisks indicate statistical significance (Mann–Whitney test). A schematic representation of the assay conditions is shown at the top.

Since we found that the role of TIP60 in suppressing 53BP1 binding to damaged DNA is not involved in fork protection conferred by TIP60 loss in BRCA-deficient cells, we sought to investigate what other activities of TIP60 may participate in this. Previously, TIP60 was found to be required for ATM activation, by catalyzing its activatory acetylation at Lys3016 [23, 30]. Similar to TIP60 depletion, knockdown of ATM suppressed HU-induced fork degradation in HeLa-BRCA2^KO^ cells (Fig. 2A; Supplementary Fig. S1C). Moreover, we observed a similar impact of ATM depletion in RPE1-BRCA1^KO^ cells, as well as in DLD1-BRCA2^KO^ cells (Fig. 2B, C; Supplementary Fig. S1C), indicating that the effect of ATM is not restricted to BRCA2, and is not cell line specific. These findings suggest that the role of TIP60 in ATM activation is responsible for its impact on fork degradation. Indeed, ATM depletion also suppressed fork slowing (Fig. 2D), suggesting that the protective effect of ATM loss on fork stability reflects defective fork reversal.

To further confirm this, we inhibited ATM using the specific inhibitor KU55933 and measured HU-induced fork degradation. In both HeLa-BRCA2^KO^ cells and RPE1-BRCA1^KO^ cells, we observed a dose-dependent suppression of HU-induced fork degradation upon treatment with KU55933 (Fig. 2E, F). This further validates a role for ATM in promoting fork degradation in BRCA-deficient cells.

**Fig. 3:**
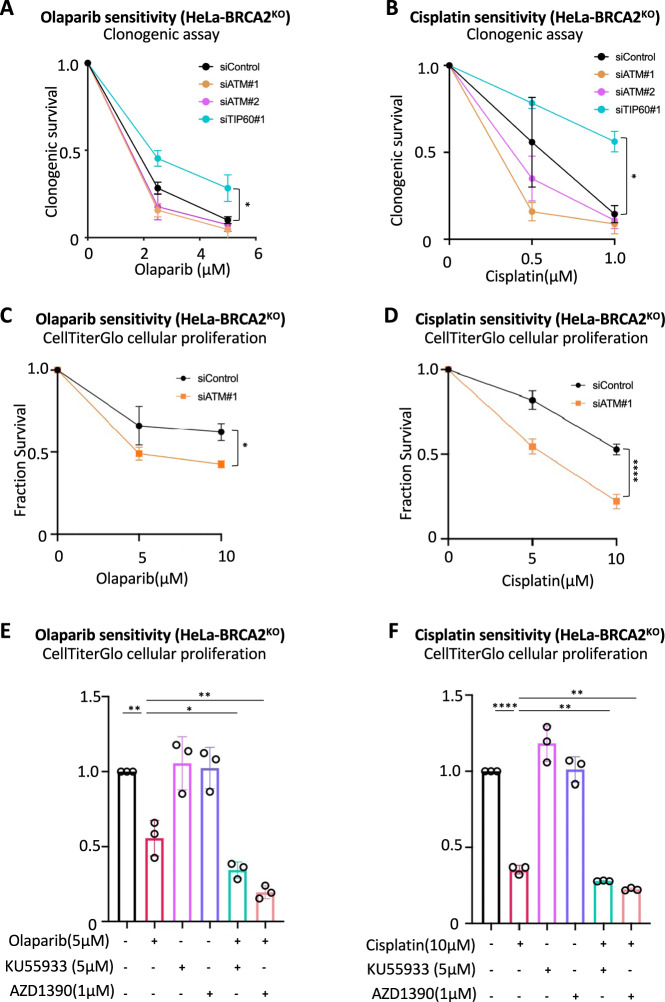
TIP60 and ATM differentially affect the chemosensitivity of BRCA2-deficient cells. **A**, **B** Clonogenic survival experiments showing that, unlike TIP60 depletion, knockdown of ATM does not promote olaparib (**A**) or cisplatin (**B**) resistance of HeLa-BRCA2^KO^ cells. The average of three experiments, with standard deviations indicated as error bars, is shown. Asterisks indicate statistical significance (2-way ANOVA). **C**, **D** CellTiterGlo cellular proliferation experiments showing that ATM depletion increases olaparib (**C**) and cisplatin (**D**) sensitivity of HeLa-BRCA2^KO^ cells. The average of three experiments, with standard deviations indicated as error bars, is shown. Asterisks indicate statistical significance (2-way ANOVA). **E**, **F** CellTiterGlo cellular proliferation experiments showing that ATM inhibition by KU55933 and AZD1390 increases olaparib (**E**) and cisplatin (**F**) sensitivity of HeLa-BRCA2^KO^ cells. The average of three experiments, with standard deviations indicated as error bars, is shown. Asterisks indicate statistical significance (*t*-test, two-tailed, unpaired).

Since fork protection promotes chemoresistance in BRCA-deficient cells [13], we investigated the impact of ATM on olaparib and cisplatin sensitivity. In contrast to loss of TIP60, which, as we previously showed [15], causes resistance to these drugs, ATM depletion did not promote resistance to cisplatin or olaparib in clonogenic assays (Fig. 3A, B). Indeed, both clonogenic assays (Fig. 3A, B) and CellTiterGlo cellular proliferation assays (Fig. 3C, D) indicated that ATM loss may in fact promote increased sensitivity of BRCA2-knockout HeLa cells to olaparib and cisplatin. In line with this, as well as with previous reports [31], ATM inhibition by two different inhibitors, KU55933 or AZD1390, also slightly increased olaparib and cisplatin sensitivity of BRCA2-deficient cells (Fig. 3E, F). Overall, these results are in line with our previous findings that the role of TIP60 in chemoresistance reflects its inhibition of 53BP1 binding to damaged DNA and subsequent NHEJ repair [15]. In addition, these results suggests that this novel ATM-dependent role of TIP60 in fork protection described here, does not contribute to chemoresistance.

### ATM promotes fork reversal by recruiting the SMARCAL1 translocase to stalled replication forks

**Fig. 4:**
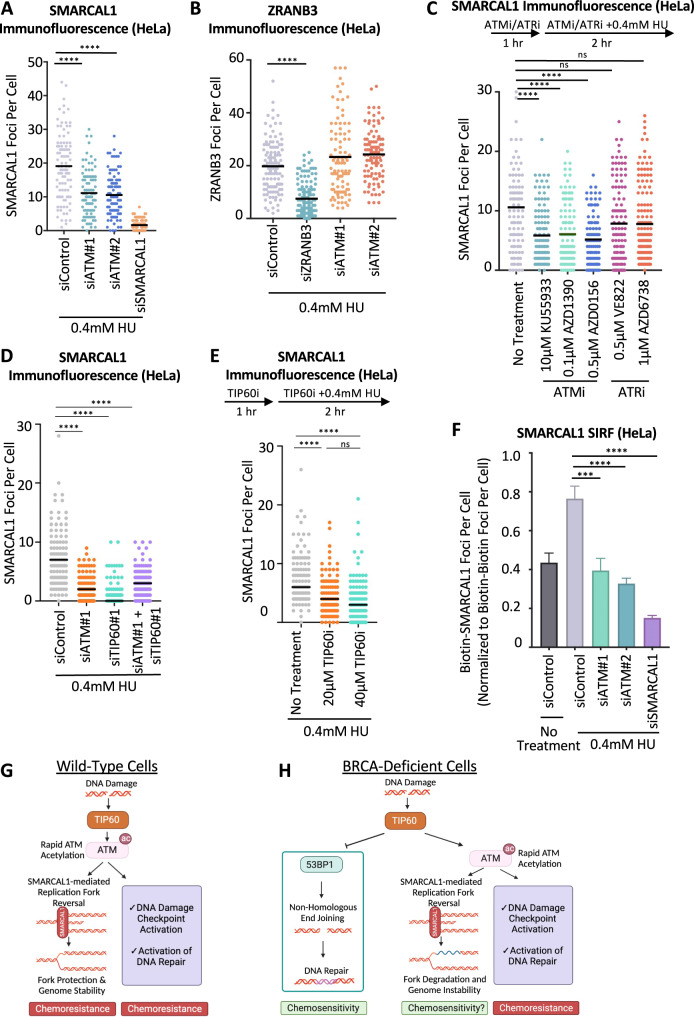
ATM activity promotes the recruitment of the SMARCAL1 translocase to stalled replication forks. **A** SMARCAL1 immunofluorescence experiment showing that ATM depletion reduces HU-induced SMARCAL1 chromatin foci formation. HeLa cells were treated with 0.4 mM HU for 2 h. SMARCAL1 depletion was used as control, to demonstrate the specificity of the immunofluorescence signal. In addition, western blots confirming the knockdown are shown in Supplementary Fig. S2C. At least 75 cells were quantified for each condition. The mean value is represented on the graph, and asterisks indicate statistical significance (*t*-test, two-tailed, unpaired). **B** ZRANB3 immunofluorescence experiment showing that ATM depletion does not affect HU-induced ZRANB3 chromatin foci formation. HeLa cells were treated with 0.4 mM HU for 2 h. ZRANB3 depletion was used as control, to demonstrate the specificity of the immunofluorescence signal. At least 75 cells were quantified for each condition. The mean value is represented on the graph, and asterisks indicate statistical significance (*t*-test, two-tailed, unpaired). **C** SMARCAL1 immunofluorescence experiment showing that ATM inhibition with three different inhibitors, but not ATR inhibition with two different inhibitors, reduces HU-induced SMARCAL1 chromatin foci formation. A schematic representation of the assay conditions is shown at the top. At least 75 cells were quantified for each condition. The mean value is represented on the graph, and asterisks indicate statistical significance (*t*-test, two-tailed, unpaired). **D**, **E** SMARCAL1 immunofluorescence experiments showing that TIP60 depletion (**D**) or TIP60 inhibition by the specific inhibitor NU9056 (**E**) reduces HU-induced SMARCAL1 chromatin foci formation, similar to ATM inactivation. At least 100 cells were quantified for each condition. The mean value is represented on the graph, and asterisks indicate statistical significance (*t*-test, two-tailed, unpaired). **F** SMARCAL1 SIRF experiment showing that ATM depletion reduces HU-induced SMARCAL1 binding to nascent DNA. HeLa cells were treated with 0.4 mM HU for 2 h or left untreated, as indicated. SMARCAL1 depletion was used as control, to demonstrate the specificity of the SIRF signal. Bars indicate the mean values, error bars represent standard errors, and asterisks indicate statistical significance (*t*-test, two-tailed, unpaired). **G**, **H** Schematic representations of the proposed model. In wild-type cells (**G**), TIP60-mediated acetylation of ATM promotes ATM activation, resulting in DNA damage checkpoint activation and DNA repair, as well as SMARCAL1 recruitment to stalled replication forks to initiate fork reversal. In BRCA-deficient cells (**H**), TIP60 promotes genomic instability through two different mechanisms: First, by acetylating histones, it inhibits 53BP1 binding to DNA breaks, thus suppressing NHEJ-mediated repair of these breaks, resulting in chemosensitivity. Second, by acetylating ATM, it promotes SMARCAL1-mediated fork reversal, which primes forks to nucleolytic degradation in these cells.

We next sought to understand why loss of ATM activity suppresses the reversal of stalled replication forks. Fork reversal is catalyzed by DNA translocases HLTF, ZRANB3, and SMARCAL1 [3–10]. We hypothesized that ATM may promote the recruitment of these translocases to stalled replication forks, thereby catalyzing fork reversal. Immunofluorescence experiments indicated that ATM depletion reduces chromatin foci of SMARCAL1 upon HU treatment, in both HeLa and DLD1 cells (Fig. 4A; Supplementary Fig. S2A–C). In contrast, ATM depletion did not reduce ZRANB3 foci under these conditions (Fig. 4B). Further confirming the specific impact of ATM on SMARCAL1 recruitment, ATM inhibition by three different inhibitors, namely KU55933, AZD1390, or AZD0156 reduced SMARCAL1 chromatin foci upon HU treatment (Fig. 4C).

To investigate if the impact of TIP60 loss on fork slowing and degradation described above (Fig. 1) is caused by deficient ATM activation, we next inactivated TIP60 and measured SMARCAL1 foci formation upon HU treatment. Similar to ATM knockdown, depletion of TIP60 resulted in reduced SMARCAL1 foci (Fig. 4D). Moreover, TIP60 inhibition using the specific inhibitor NU9056 also reduced SMARCAL1 foci (Fig. 4E). Importantly, co-depletion of ATM and TIP60 did not further reduce SMARCAL1 foci (Fig. 4D), suggesting that ATM and TIP60 act in the same pathway to promote SMARCAL1 recruitment to stalled replication forks.

Since assaying chromatin foci in immunofluorescence experiments does not directly investigate protein recruitment to replication forks, we sought to specifically measure SMARCAL1 binding to nascent DNA. To this end, we employed the SIRF (in situ analysis of protein interactions at replication forks) assay [32], a proximity ligation assay, to measure the recruitment of SMARCAL1 to EdU-labeled nascent DNA. HU treatment resulted in an increase in SMARCAL1 SIRF foci in HeLa cells, and SMARCAL1 depletion suppressed these foci (Fig. 4F), showing that this assay is able to specifically measure SMARCAL1 binding to stalled replication forks. In line with the immunofluorescence experiments, ATM depletion significantly suppressed SMARCAL1 SIRF foci (Fig. 4F), thus further confirming that ATM is required for SMARCAL1 recruitment to stalled replication forks, to catalyze their reversal. Overall, these findings suggest that TIP60-mediated activation of ATM promotes fork reversal by SMARCAL1 (Fig. 4G). In BRCA-deficient cells, this fork reversal enables fork degradation (Fig. 4H). ATM loss increases the chemosensitivity of BRCA-deficient cells, while loss of TIP60 induces chemoresistance in BRCA2-deficient cells; this chemoresistance requires 53BP1, suggesting it occurs through NHEJ-mediated DNA repair.

It was previously shown that ATR phosphorylates SMARCAL1 at Ser652, and this does not affect SMARCAL1 binding to stalled forks, but instead it may suppress its fork reversal activity [33]. Indeed, immunofluorescence experiments showed that ATR inhibition using two different inhibitors, namely VE822 or AZD6738, does not reduce SMARCAL1 chromatin foci upon HU treatment to the same extent as ATM inhibition (Fig. 4C). Moreover, unlike ATM knockdown, depletion of ATR did not reduce SMARCAL1 SIRF foci upon HU treatment (Supplementary Fig. S3A, B), overall indicating that ATM, but not ATR, specifically regulates SMARCAL1 recruitment to stalled replication forks. In line with this, knockdown of ATR did not overtly affect fork slowing (Supplementary Fig. S3C), and did not restore fork protection to BRCA2-deficient cells to the extent of ATM depletion (Supplementary Fig. S3D).

### CRISPR screens reveal genetic determinants of cellular sensitivity to multiple ATM inhibitors in wild-type cells and BRCA-deficient cells

Previous studies [31] and our own results described above (Fig. 3) indicated that ATM inhibition may potentially be employed to enhance the chemosensitivity of cancer cells. This may be particularly relevant for BRCA-deficient cells, in light of our findings that ATM promotes fork degradation in these cells (Fig. 2), since previous studies found that fork stability is essential for the viability of BRCA-deficient cells [13, 34]. Indeed, ATM inhibitors are currently being investigated in phase I clinical trials. Identification of genetic determinants of the cellular sensitivity to DNA repair inhibitors can provide biomarkers for better deployment of these drugs in the clinic, as well as reveal insights into DNA repair mechanism and regulation [14]. Prompted by the unexpected role of ATM we uncovered in fork slowing and degradation in BRCA-deficient cells, we performed a series of genome-wide CRISPR knockout screens to identify genes that cause differential sensitivity to multiple ATM inhibitors in wildtype and BRCA2^KO^ HeLa cells.

**Fig. 5:**
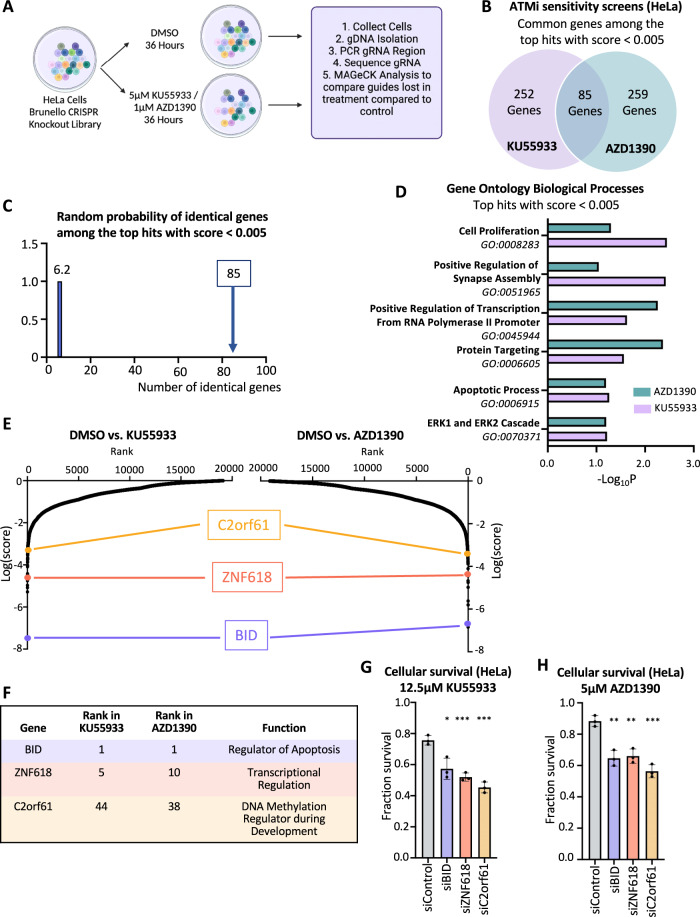
Genome-wide CRISPR knockout screens identify common genes which regulate the cellular sensitivity to multiple ATM inhibitors. **A** Overview of the CRISPR knockout screens to identify genes that regulate the sensitivity to the ATM inhibitors KU5933 and AZD1390 in HeLa cells. **B** Diagram showing the overlap of identical genes within the top hits from both ATMi screens with MAGeCK score < 0.005. **C** The number of common genes within the top hits with MAGeCK score < 0.005 (namely 85) is much higher than the random probability of identical hits, which is 6.2. **D** Biological pathways that were significantly enriched within the top hits with MAGeCK score < 0.005 from both ATMi screens using Gene Ontology Biological Processes (GO_BP) analysis. **E** Scatterplots showing the results of the ATMi sensitivity CRISPR knockout screens in HeLa cells. Each gene targeted by the library was ranked according to the MAGeCK score. Three top hits common between the two ATMi screens, chosen for subsequent validation, are indicated. **F** Table showing the ranks in each of the screens, and the biological functions of the three top common hits chosen for subsequent validation. **G**, **H** Cellular survival assays showing that knockdown of BID, ZNF618, or C12orf61 in HeLa cells results in sensitivity to the ATM inhibitors KU5933 (**G**) and AZD1390 (**H**). The average of three experiments is shown. Error bars represent standard deviations, and asterisks indicate statistical significance (*t*-test, two-tailed, unpaired). Western blots confirming the knockdown are shown in Supplementary Fig. S4A, B.

First, we infected wild-type HeLa cells with the Brunello genome-wide knockout library [35] which targets 19,114 human genes with an average of 4 guide RNAs (gRNAs) for each gene, for a total of with 76,441 unique gRNAs. Taking care to maintain 250x fold library coverage at all times, we treated library-infected cells with DMSO, or with two different ATM inhibitors, namely KU55933 and AZD1390, using low drug doses that we previously determined to reduce survival by about 25% compared to the DMSO control (Fig. 5A). After 36 h, surviving cells were collected and genomic DNA was extracted. The gRNA sequences were PCR-amplified and identified by Illumina sequencing. Bioinformatic analyses using the MAGeCK algorithm [36] were used to generate separate ranking lists of genes that were lost in the KU55933 or AZD1390 conditions compared to the control (Supplementary Table S1). This represents genes that, when inactivated, confer sensitivity to ATMi.

We found a large overlap between the KU55933 and AZD1390 screen results, indicating common response mechanisms to the two different ATMi, and highlighting the specificity of the inhibitors. Of the top hits of each of the two ATMi screens with MAGeCK negative score < 0.005 (337 genes for the KU55933 screen and 344 genes for the AZD1390 screen), 85 were present in both of them (Fig. 5B, Supplementary Table S2), which is much higher than the random probability (Fig. 5C). Biological pathways analysis of the top hits of both screens with MAGeCK negative score < 0.005 revealed biological processes common between the top hits in the two screens, including apoptosis, transcription, and cell proliferation (Fig. 5D). We next proceeded to validate three of the top common hits between the two ATMi screens, namely BID, ZNF618, and C2orf61 (Fig. 5E, F). Knockdown of each of these top hits resulted in sensitivity to both KU55933 and AZD1390 (Fig. 5G, H, Supplementary Fig. S4A, B), thereby validating our screen results.

**Fig. 6:**
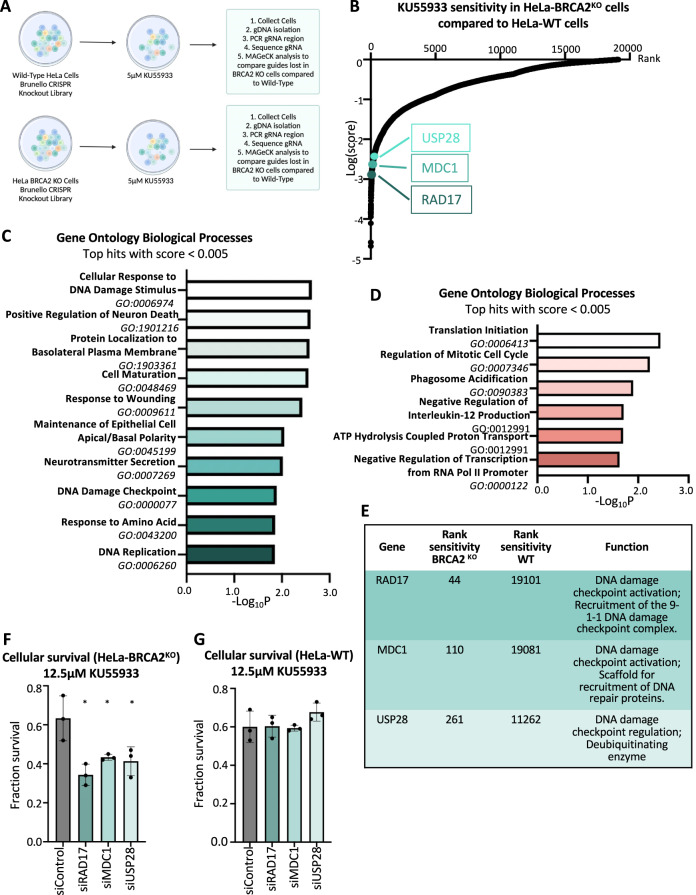
Genome-wide CRISPR knockout screens identify a differential set of genetic determinants of cellular sensitivity to the ATM inhibitor KU55933 in wildtype compared to BRCA2-knockout HeLa cells. **A** Overview of the CRISPR knockout screens to identify genes that differentially regulate the sensitivity of wiltdype and BRCA2-knockout HeLa cells to KU55933. **B** Scatterplot showing the results of genome-wide CRISPR knockout screens for cellular sensitivity to the ATM inhibitor KU55933 in wildtype and BRCA2-knockout HeLa cells. Each gene targeted by the library was ranked according to the MAGeCK score indicating genes which, when inactivated, cause specific sensitivity to HeLa-BRCA2^KO^ cells compared to wild-type HeLa cells. Top hits chosen for validation are indicated. **C** Gene Ontology analysis of the top hits with a score <0.005 which cause specific KU55933 sensitivity to BRCA2-knockout compared to wild-type HeLa cells. GO_BP terms with a negative logP of 1.85 or lower are shown. **D** Gene Ontology analysis of the top hits with a score < 0.005 which cause specific KU55933 sensitivity to wildtype compared to BRCA2-knockout HeLa cells. GO_BP terms with a negative logP of 1.6 or lower are shown **E**. Table showing the ranks, and the biological functions of the hits chosen for subsequent validation. **F**, **G** Cellular survival assays showing that knockdown of RAD17, MDC1, or USP28 results in KU55933 sensitivity in HeLa-BRCA2^KO^ cells (**F**), but not in wild-type HeLa cells (**G**). The average of three experiments is shown. Error bars represent standard deviations, and asterisks indicate statistical significance (*t*-test, two-tailed, unpaired). Western blots confirming the knockdown are shown in Supplementary Fig. S4C–E.

Next, we infected HeLa-BRCA2^KO^ cells with the same library, and performed a KU55933 sensitivity screen using the same conditions of the KU55933 sensitivity screen in wild-type cells described above (Fig. 6A). We then compared the gRNA representation in the BRCA2-knockout KU55933-treated sample to the wild-type KU55933-treated sample (Fig. 6B; Supplementary Table S3). This allowed us to identify genes which, when inactivated, confer differential ATMi sensitivity to BRCA2-knockout cells compared to wild-type cells. Pathway analyses of the top hits which specifically cause sensitivity in BRCA2^KO^ cells compared to wild-type cells, revealed that DNA repair processes were enriched among the genetic pathways to which these top hits are assigned (Fig. 6C). In contrast, when we analyzed genes whose depletion specifically caused sensitivity to wildtype compared to BRCA2^KO^ cells, pathway analyses revealed a different distribution of biological processes, with transcription and translation as notable enriched processes (Fig. 6D). Overall, these results indicate a differential response of wildtype and BRCA2-deficient cells to ATM inhibition, and suggest that DNA repair is the major mechanism needed for survival of ATMi-treated BRCA2^KO^ cells. To confirm this, we sought to validate three representative DNA repair genes scoring within the top hits (MAGeCK negative score < 0.005), namely RAD17, MDC1, and USP28 (Fig. 6B, E). Knockdown of any of these genes conferred ATMi sensitivity to BRCA2-knockout, but not to wild-type HeLa cells (Fig. 6F, G, Supplementary Fig. S4C–E), thereby validating our screens, and establishing an essential role for DNA repair pathways in specifically mediating the resistance of BRCA2-deficient cells to ATM inhibitors.

## Discussion

Protection of stalled replication forks is an important component of genome stability maintenance. Upon encountering fork-blocking structures, replication forks can be reversed by annealing of the nascent strands of the sister chromatids, an event catalyzed by DNA translocases such as SMARCAL1, ZRANB3, and HLTF [3–10]. In wild-type cells, the BRCA pathway protects the integrity of reversed forks by loading RAD51 on the reversed arm, thereby protecting it against nucleolytic degradation by MRE11 and other nucleases. In contrast, in BRCA-deficient cells, the inability to load RAD51 renders the reversed arm susceptible to nucleolysis [11, 12, 37]. Nucleolytic degradation of stalled replication forks in BRCA-deficient cells can results in genomic instability [37]. However, under certain circumstances, suppression of fork degradation is associated with chemoresistance of BRCA-deficient cells [13]. Since fork reversal is a prerequisite for fork degradation, blocking fork reversal protects against fork degradation in these cells [3–10]. However, the impact of fork reversal on chemoresistance is nevertheless unclear [6]. Here, we show that the TIP60-ATM axis promotes fork reversal through SMARCAL1 recruitment to stalled replication forks. In BRCA-deficient cells, this fork reversal results in fork degradation. While we only investigated the impact of TIP60-ATM loss in BRCA1- or BRCA2-deficient cells, it is likely that fork protection is also restored in cells deficient in other BRCA pathway components required for fork protection, such as BARD1 [38] and PALB2 [39].

We previously showed that loss of TIP60 induces chemoresistance in BRCA2-deficient cells [15]. Mechanistically, we showed that this requires the presence of 53BP1, which promotes NHEJ-mediated DNA repair. TIP60 was shown to suppress 53BP1 binding to DNA breaks, by acetylating histones at DNA damage sites forming a chromatin structure that blocks 53BP1 interaction [16, 17]. We proposed that, in the absence of TIP60, enhanced 53BP1 binding to DNA lesions promotes their NHEJ-mediated repair, thus explaining the chemoresistance observed under these conditions. We had also found at that time, that TIP60 loss also promotes fork protection in BRCA2-deficient cells. The mechanisms employed, and the relevance of this fork protection for chemoresistance, remained unclear. Here, we revisited these findings to investigate if 53BP1 is also involved in fork protection upon TIP60 loss. We found that co-depletion of 53BP1 did not restore fork degradation to TIP60-depleted BRCA2-knockout cells (Fig. 1), suggesting a 53BP1-independent mechanism, and thus different than what we previously reported for chemoresistance. Outside of histones, TIP60 also acetylates ATM, which is important for ATM activation [23, 30]. This prompted us to investigate if TIP60 activity in fork degradation involves ATM. Indeed, loss or inhibition of ATM caused fork protection, similarly to TIP60 knockdown (Fig. 2). Moreover, we showed that this fork protection is underlain by a defect in fork reversal (Fig. 2). Mechanistically, we found that ATM promotes fork reversal by recruiting the SMARCAL1 translocase to stalled replication forks (Fig. 4).

ATM has well-documented roles in promoting genomic stability in wild-type cells, including: DNA damage checkpoint activation, cell cycle control, transcriptional regulation of DNA repair, regulation of the R-loops metabolism and others [18–22]. These mechanisms are likely to be operational in BRCA-deficient cells, and promote DNA repair and genomic stability in these cells. Moreover, recent findings indicated that ATM may promote residual homologous recombination (HR)-mediated DNA repair in BRCA-deficient cells [40, 41]. These functions of ATM potentially explain why its loss increases the chemosensitivity of BRCA-deficient cells. Since ATM is activated by TIP60 [23, 30], loss of TIP60 also causes the defects observed upon ATM loss. However, in addition to activating ATM, TIP60 also suppresses 53BP1-mediated NHEJ [15–17]. Thus, upon loss of TIP60, genomic stability of BRCA-deficient cells is enhanced, since NHEJ repair is activated [15]. Our experiments (Fig. 3) indicate that the net result of TIP60 inactivation in BRCA-deficient cells is chemoresistance, suggesting that the activation of 53BP1-mediated DNA repair overcomes the DNA repair deficiency caused by the suppression of ATM activation. Overall, this model explains the differential effect of TIP60 and ATM on the chemosensitivity of BRCA-deficient cells (Fig. 4G).

As mentioned above, our findings suggest that regulation of DSB repair, rather than fork degradation, is the relevant activity involved in TIP60-mediated chemosensitivity in BRCA2-deficient cells. Nevertheless, a role for fork protection mediated by loss of the TIP60-ATM pathway in chemoresistance cannot be ruled out. Interestingly, a recent publication [42] showed that ATM promotes MRE11-mediated processing of post-replicative single stranded DNA gaps left unrepaired behind the replication fork in HR-deficient cells. Thus, this may represent yet another mechanism through which the TIP60-ATM axis promotes genomic instability in BRCA-deficient cells. What is the relative contribution to ATM-mediated fork degradation of the promotion of SMARCAL1-catalyzed fork reversal described here, versus this MRE11-mediated gap expansion previously described, remains to be determined.

Perhaps surprisingly, our data indicate that this activity of ATM in fork reversal is not shared by ATR (Supplementary Fig. S3), even though ATR is traditionally viewed as the replication stress checkpoint kinase. Indeed, it was previously shown that ATR phosphorylates SMARCAL1 and this does not impact SMARCAL1 recruitment, but instead it suppresses its activity in fork reversal [33]. Overall, these findings suggest that SMARCAL1 activity is differentially regulated by ATM and ATR: ATM promotes its recruitment to stalled replication forks, while ATR inhibits its activity once recruited to stalled forks.

Finally, we performed a series of genome-wide CRISPR knockout genetic screens to identify genetic determinants of the sensitivity to ATMi in wildtype and BRCA2-deficient cells (Figs. 5, 6). We provide a comprehensive set of genes whose inactivation sensitizes cells to multiple ATMi, which may thus represent biomarkers of the tumor cells’ response to ATM inhibition. Since ATM inhibitors are making their way in the clinic, this dataset may eventually prove a valuable resource. Interestingly, we observed that the genetic determinants of ATMi sensitivity were markedly different between wildtype and BRCA-deficient cells. In BRCA2-knockout cells, genes involved in DNA repair were essential for survival upon ATMi treatment, which was not the case in wild-type cells (Fig. 6). This may potentially be related to the role of the TIP60-ATM axis in fork degradation in BRCA-deficient cells identified here, since previous studies found that fork stability is essential for viability of BRCA-deficient cells [13, 34]. In any case, this differential set of genetic vulnerabilities may eventually help guide the rational deployment of ATM inhibitors for treatment of BRCA-deficient tumors.

## Materials and methods

### Cell culture and protein techniques

Human HeLa and RPE1 cells were cultured in DMEM supplemented with 10% fetal calf serum and 1% Pen/Strep. DLD1 cells were cultured in RPMI supplemented with 10% fetal calf serum and 1% Pen/Strep. HeLa-BRCA2^KO^ cells were generated in our laboratory [43]. DLD1-BRCA2^KO^ cells (Horizon HD105-007) were obtained from Dr. Robert Brosh (National Institute on Aging, Baltimore, MD). RPE1-BRCA1^KO^ (also harboring p53 homozygous deletion) were obtained from Dr. Alan D’Andrea (Dana-Farber Cancer Institute, Boston, MA) [44].

Gene knockdown was performed using Lipofectamine RNAiMAX (ThermoFisher). AllStars negative control siRNA (Qiagen 1027281) was employed as control. The following oligonucleotide sequences (Stealth or SilencerSelect siRNA, ThermoFisher) were used: 53BP1: TCCCAGAGTTGATGTTTCTTGTGAA; TIP60#1: GATGGACGTAAGAACAAGAGTTATT; TIP60#2: CACCCATTCATCCAGACGTTTGTTG; ZRANB3: TGGCAATGTAGTCTCTGCACCTATA; SMARCAL1: CACCCTTTGCTAACCCAACTCATAA; ATM#1: AM51331; ATM#2: s1708; ATR#1: AM16708; ATR#2: s227305; BID: s1985; ZNF618: s41743; C2orf61: s49807; MDC1: s18579; RAD17: s11723; USP28: s33509.

Denatured whole cell extracts were prepared by boiling cells in 100 mM Tris, 4% SDS, 0.5 M β-mercaptoethanol. Antibodies used for western blots were: Vinculin (Santa Cruz sc-25336), GAPDH (Santa Cruz sc-47724), TIP60 (Santa Cruz sc-166323), ATM (Cell Signaling 2873 S), SMARCAL1 (Santa Cruz sc-376377), 53BP1 (Bethyl A300-272A), ZRANB3 (Bethyl A303-033A), ATR (Cell Signaling 2790 S), C2orf61 (Novus NBP2-38676), BID (R&D MAB860), RAD17 (Proteintech 13358-1-AP), USP28 (Novus NB110-40543), MDC1 (Novus NB100-395).

Small molecule inhibitors used were: ATM inhibitors (KU55933, Selleck S1092; AZD1390, Selleck S8680; AZD0156, Selleck S8375), ATR inhibitors (VE822, Selleck S7102; AZD6738, Selleck S7693), and TIP60 inhibitors (NU9056, Tocris 4903).

### Drug sensitivity assays

For clonogenic assays, after 2 days of siRNA treatment, 500 cells were plated into 6-well plates and treated with the indicated doses of olaparib or cisplatin. After 3 days of treatment, media was replaced. After 14 days, colonies were fixed and stained with 2% crystal violet. For cellular survival assays, after 2 days of siRNA treatment, 250,000 cells were plated in two wells of a 6-well plate. One well remained untreated and the other well was treated with the indicated doses of AZD1390 or KU55933. After 3 days of treatment, cells were counted using the EVE automated cell counter (NanoEntek), and the cell survival fraction was calculated. CellTiterGlo cellular proliferation assays were performed using the CellTiterGlo reagent (Promega G7572) according to the manufacturer’s instructions. For each condition, 1500 cells were plated into 96-well plates and treated as indicated. Three days later, CellTiterGlo reagent was added for 10 min and the luminescence was read on a plate reader.

### Immunofluorescence

Cells were seeded onto 4-chamber glass sides and fixed with 3.7% paraformaldehyde for 10 min, followed by two PBS washes. Cells were then permeabilized with 0.3% Triton X-100 for 10 min. After two PBS washes, slides were blocked with 5% BSA and 0.1% Triton in PBS for 30 min, followed by incubation with the primary antibody diluted in 3% BSA in PBS, for 1 h at room temperature. After three washes with PBS, the secondary antibody was added for 1 h. Slides were mounted with DAPI-containing Vectashield mounting medium (Vector Labs). Primary antibodies used for immunofluorescence were: SMARCAL1 (Santa Cruz sc-376377); ZRANB3 (Bethyl A303-033A). Slides were imaged using a Leica SP5 confocal microscope. The number of foci/nucleus was quantified using ImageJ 1.52p software. At least 100 cells were quantified for each sample.

### In situ analysis of protein interactions at replication forks (SIRF)

After siRNA treatment for 2 days, HeLa cells were seeded into 8-chamber slides and 24 h later they were pulse-labeled with 50 µM EdU for 10 min followed by 0.4 mM HU for 2 hr as indicated. Cells were permeabilized with 0.5% Triton for 10 min at 4 C, washed with PBS, fixed at room temperature with 3% paraformaldehyde in PBS for 10 min, washed again in PBS, and then blocked in 3% BSA in PBS for 30 min. Cells were then subjected to Click-iT reaction with biotin-azide using the Click-iT Cell Reaction Buffer Kit (ThermoFisher, C10269) for 30 min and incubated overnight at 4 C with primary antibodies diluted in PBS with 1% BSA. The primary antibodies used were: Biotin (mouse: Jackson ImmunoResearch 200-002-211; rabbit: Bethyl Laboratories A150-109A); SMARCAL1 (Santa Cruz sc-376377). Next, cells were subjected to a proximity ligation reaction using the Duolink kit (Millipore Sigma) according to the manufacturer’s instructions. Slides were imaged using a Leica SP5 confocal microscope and images were analyzed using ImageJ 1.52p software. At least 100 cells were quantified for each sample. For each sample, the number of SMARCAL1-EdU foci were divided by the average of the number of Biotin-Biotin foci for that respective sample.

### CRISPR screens

For CRISPR knockout screens, the Brunello Human CRISPR knockout pooled lentiviral library (Addgene 73179) was used [35]. This library is comprised of 76,411 gRNAs that target 19,114 genes. Fifty million wild-type HeLa cells and fifty million HeLa-BRCA2^KO^ cells were infected with this library at a multiplicity of infection (MOI) of 0.4 to achieve 250-fold coverage and selected for 4 days with 0.6 μg/mL puromycin. Twenty million library-infected cells (to maintain 250-fold coverage) were used for each drug condition: DMSO (vehicle control), 5 μM KU55933 (Selleck S1092), and 1 μM AZD1390 (Selleck S8680). Cells were treated for 72 h and then collected. Compared to control cells, survival of ATMi-treated cells in wild-type HeLa cells was 68% for KU55933 and 68% for AZD1390, respectively. Survival of KU55933-treated HeLa-BRCA2^KO^ cells was 78% compared to control. Genomic DNA was isolated using the DNeasy Blood and Tissue Kit (Qiagen 69504) per the manufacturer’s instructions. Genomic DNA from twenty million cells (corresponding to the equivalent of 250-fold library coverage) was employed for PCR using Illumina adapters to identify the gRNA representation in each sample. 10 μg of gDNA was used in each PCR reaction along with 20 μl 5X HiFi Reaction Buffer, 4 μl of P5 primer, 4 μl of P7 primer, 3 μl of Radiant HiFi Ultra Polymerase (Stellar Scientific), and water. The P5 and P7 primers were determined using the user guide provided with the CRISPR libraries (https://media.addgene.org/cms/filer_public/61/16/611619f4-0926-4a07-b5c7-e286a8ecf7f5/broadgpp-sequencing-protocol.pdf). The PCR cycled as follows: 98 °C for 2 min before cycling, then 98 °C for 10 s, 60 °C for 15 s, and 72 °C for 45 s, for 30 cycles, and finally 72 °C for 5 min. After PCR purification, the final product was Sanger sequenced to confirm that the guide region is present, followed by qPCR to determine the exact amount of PCR product present. The purified PCR product was then sequenced with Illumina HiSeq 2500 single read for 50 cycles, targeting ten million reads. Next, the sequencing results were analyzed bioinformatically using the MAGeCk algorithm, which takes into consideration raw gRNA read counts to test if individual guides vary significantly between the conditions [36]. The MAGeCK software and instructions on running it were obtained from https://sourceforge.net/p/mageck/wiki/libraries/. Finally, analyses of the Gene Ontology pathways enriched among the top hits was performed using DAVID [45, 46].

### DNA fiber combing

Cells were treated with siRNA as indicated, for 2 days. For fiber degradation assays, cells were incubated with 100 μM IdU for 30 min, washed with PBS and incubated with 100 μM CldU for another 30 min. Cells were washed and then treated with 4 mM HU for 4 h. For fiber slowing assays, cells were incubated with 100 μM IdU for 30 min, washed with PBS and incubated with 100 μM CldU in combination with 0.4 mM HU for 2 h. Cells were harvested and processed using the FiberPrep kit (Genomic Vision EXT-001) according to the manufacturer’s instructions. DNA molecules were stretched onto coverslips (Genomic Vision COV-002-RUO) using the FiberComb Molecular Combing instrument (Genomic Vision MCS-001). Slides were incubated with primary antibodies (Abcam 6326 for detecting CIdU; BD 347580 for detecting IdU; Millipore Sigma MAB3034 for detecting DNA), washed with PBS and incubated with Cy3, Cy5, or BV480-coupled secondary antibodies (Abcam 6946, Abcam 6565 and BD Biosciences 564879). Following mounting, slides were imaged using a Leica SP5 confocal microscope. At least 100 tracts were quantified for each sample.

### Statistical analyses

For drug sensitivity assays, immunofluorescence, and SIRF assays, the *t*-test (two-tailed, unpaired) was used (unless otherwise indicated in the figure legends). For the DNA fiber assay, the Mann–Whitney statistical test was performed. Statistical significance is indicated for each graph (ns = not significant, for *P* > 0.05; *P ≤ 0.05; **P ≤ 0.01; ***P ≤ 0.001, ****P ≤ 0.0001). All source data underlying each of the figures, including the values plotted in graphs and the exact *p*-values are presented in the Supplementary Table S4. The random probability of identical genes within the top hits with MAGeCK score < 0.005 was calculated by multiplying the individual probabilities of each set: [(number of genes in set 1/total number of genes in the library) * (number of genes in set 2/total number of genes in the library)].

## Supplementary information

Supplementary Material.

Supplementary Table S1

Supplementary Table S2

Supplementary Table S3

Supplementary Table S4

## Data Availability

All data generated or analyzed during this study are included in this published article and its supplementary information files, or available from the authors upon reasonable request. The MAGeCK files showing the complete CRISPR screening datasets are presented in the Supplementary Tables S1 and S3. All source data underlying each of the figures, including the values plotted in graphs, the exact *p*-values, and the uncropped blots are presented in the Supplementary Table S4.
