# Supplementary material for: The TIP60-ATM axis regulates replication fork stability in BRCA-deficient cells
Source: Oncogenesis. 2022 Jun 18;11(1):33. doi: 10.1038/s41389-022-00410-w (PMC9206655; doi:10.1038/s41389-022-00410-w)

Legends to Supplementary Tables and Figures

Supplementary Tables

Supplementary Table S1. List of all genes in the CRISPR knockout ATMi sensitivity screens ranked by MAGeCK score.

Supplementary Table S2. List of common hits with MAGeCK score lower than 0.005 from the CRISPR knockout screens for sensitivity to the ATM inhibitors KU5933 and AZD1390.

Supplementary Table S3. MAGeCK analyses of the screens identifying differential genetic determinants to KU5933 in wildtype compared to BRCA2-knockout HeLa cells.

Supplementary Table S4. The source data underlying each of the figure panels, including: the values plotted in graphs, the exact p-values, and the uncropped blots.

Supplementary Figures

Supplementary Figure S1. Confirmation of gene knockdowns.

Western blots showing the depletion of TIP60 (**A**), ZRANB3 and 53BP1 (**B**), and ATM (**C**) in HeLa cells.

Supplementary Figure S2. ATM depletion reduces the recruitment of SMARCAL1 to HU-induced chromatin foci.

A, B. SMARCAL1 immunofluorescence experiments showing that ATM depletion reduces HU-induced SMARCAL1 chromatin foci formation. HeLa (**A**) and DLD1 (**B**) cells were treated with 0.4mM HU for 2 hours. SMARCAL1 depletion was used as control, to demonstrate the

specificity of the immunofluorescence signal. At least 75 cells were quantified for each condition. The mean value is represented on the graph, and asterisks indicate statistical significance (t-test, two-tailed, unpaired). **C**. Western blots showing the depletion of SMARCAL1 in HeLa cells.

Supplementary Figure S3. ATR depletion does not impair SMARCAL1-mediated fork reversal.

A. SMARCAL1 SIRF experiment showing that ATM depletion, but not ATR depletion, reduces HU-induced SMARCAL1 binding to nascent DNA. HeLa cells were treated with 0.4mM HU for 2 hours. At least 75 cells were quantified for each condition. Bars indicate the mean values, error bars represent standard errors, and asterisks indicate statistical significance (t-test, two-tailed, unpaired). **B**. Western blots showing the depletion of ATR in HeLa cells. **C**. DNA fiber combing assays showing that ATM depletion, but not ATR depletion, impairs HU-induced fork slowing in HeLa cells. Depletion of the translocase SMARCAL1 which catalyzes fork reversal is used as a control, since only reversed forks are subjected to degradation. The ratio of CldU to IdU tract lengths is presented, with the median values marked on the graph and listed at the top. At least 100 tracts were quantified for each sample. Asterisks indicate statistical significance (Mann-Whitney test). A schematic representation of the assay conditions is shown at the top. **D**. DNA fiber combing assays showing that ATM depletion, but not ATR depletion, suppresses HU-induced fork degradation in HeLa-BRCA^{KO} cells. Depletion of the translocase SMARCAL1 which catalyzes fork reversal is used as a control, since only reversed forks are subjected to degradation. The ratio of CldU to IdU tract lengths is presented, with the median values marked on the graph and listed at the top. At least 100 tracts were quantified for each sample. Asterisks indicate statistical significance (Mann-Whitney test). A schematic representation of the assay conditions is shown at the top.

Supplementary Figure S4. Confirmation of gene knockdowns.

Western blots showing the depletion of C2orf61 (**A**), BID (**B**), RAD17 (**C**), MDC1 (**D**), and USP28 (**E**) in HeLa cells.

Supplementary Figure S1

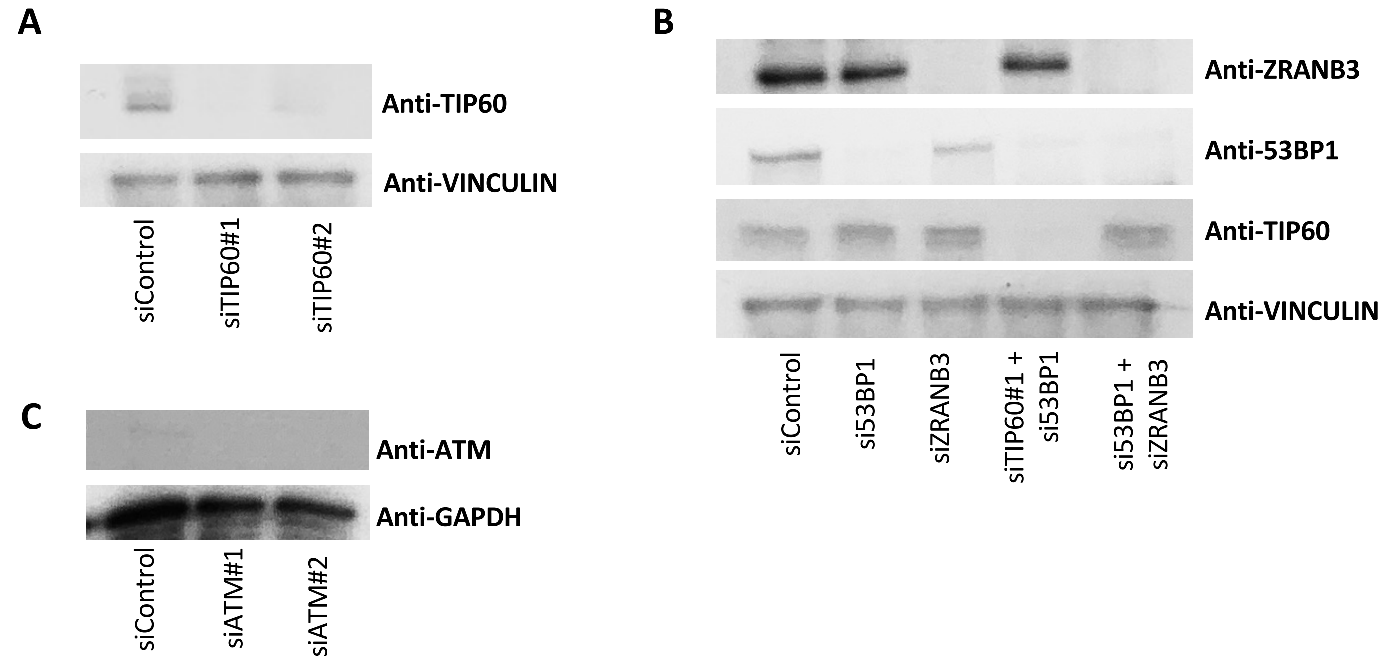

Supplementary Figure S2

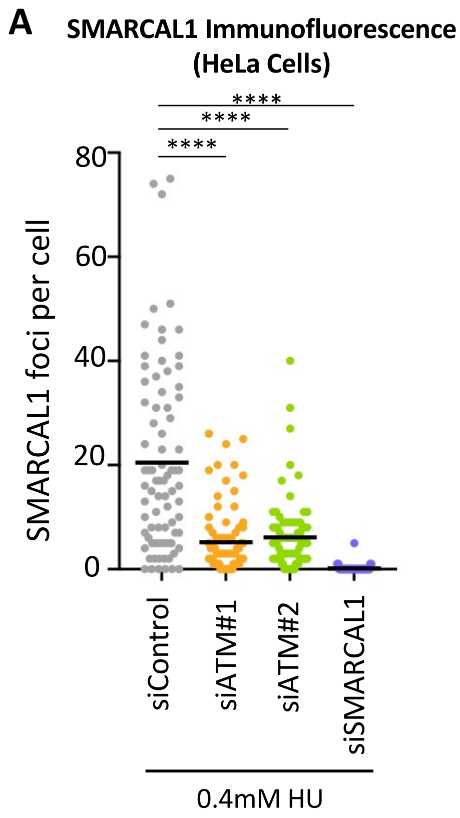

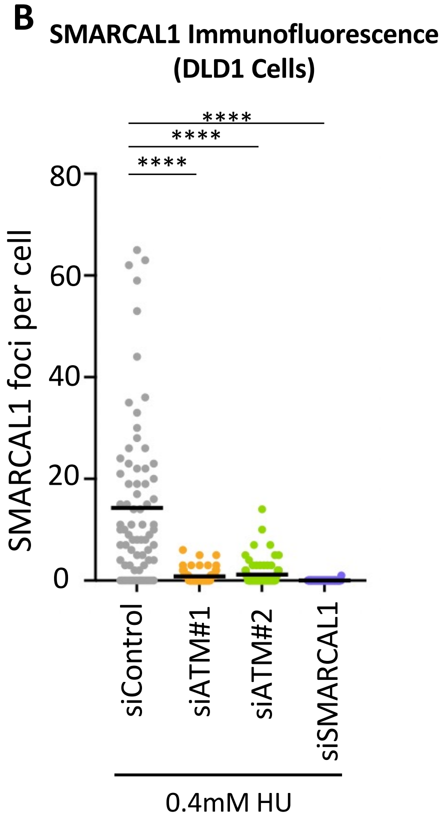

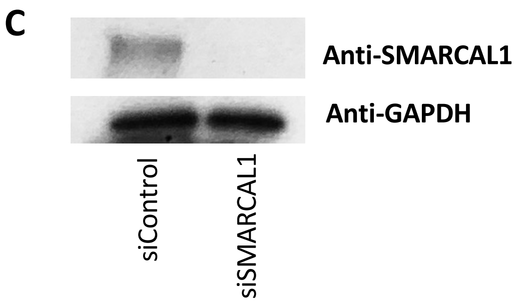

Supplementary Figure S3

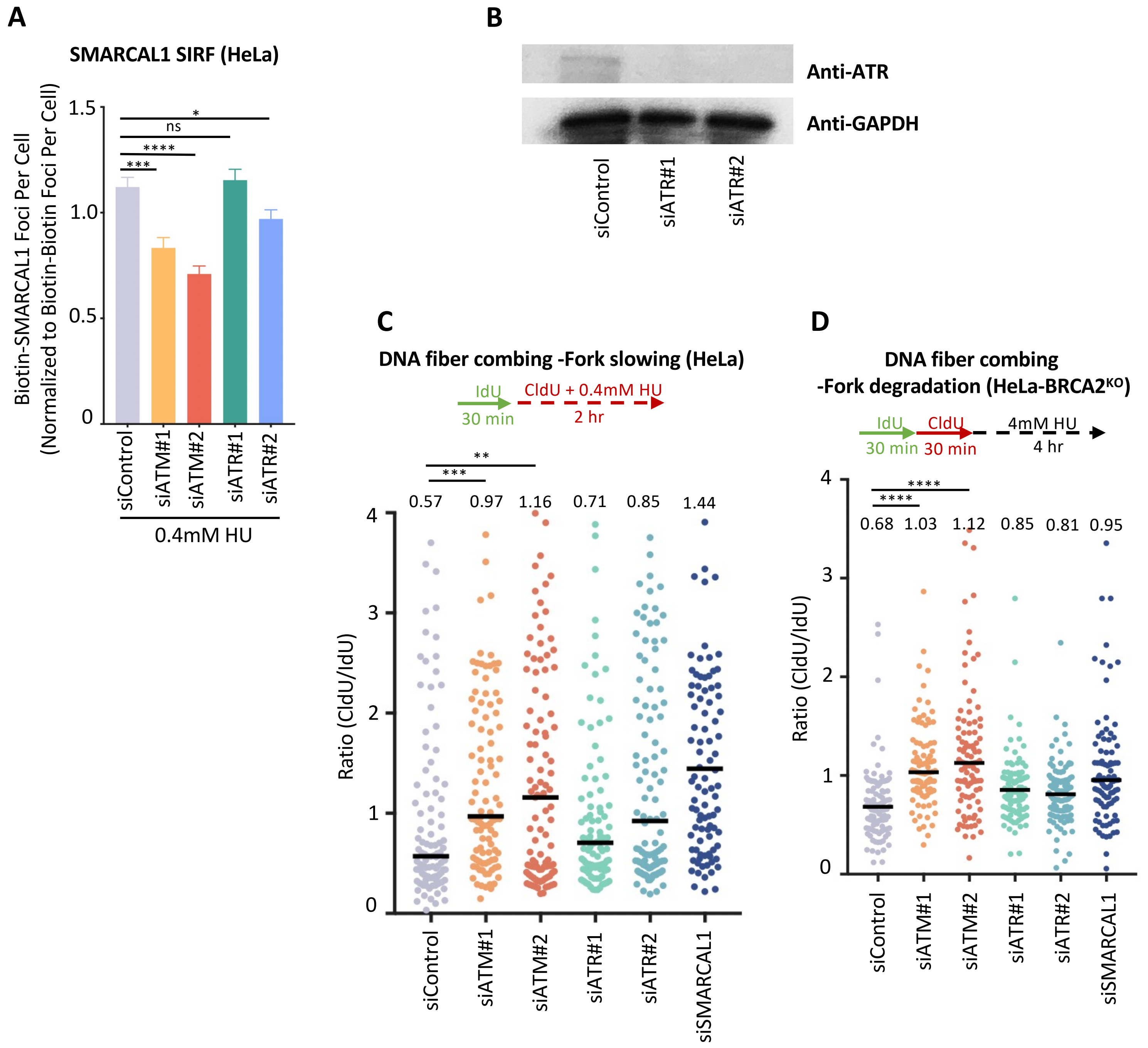

Supplementary Figure S4

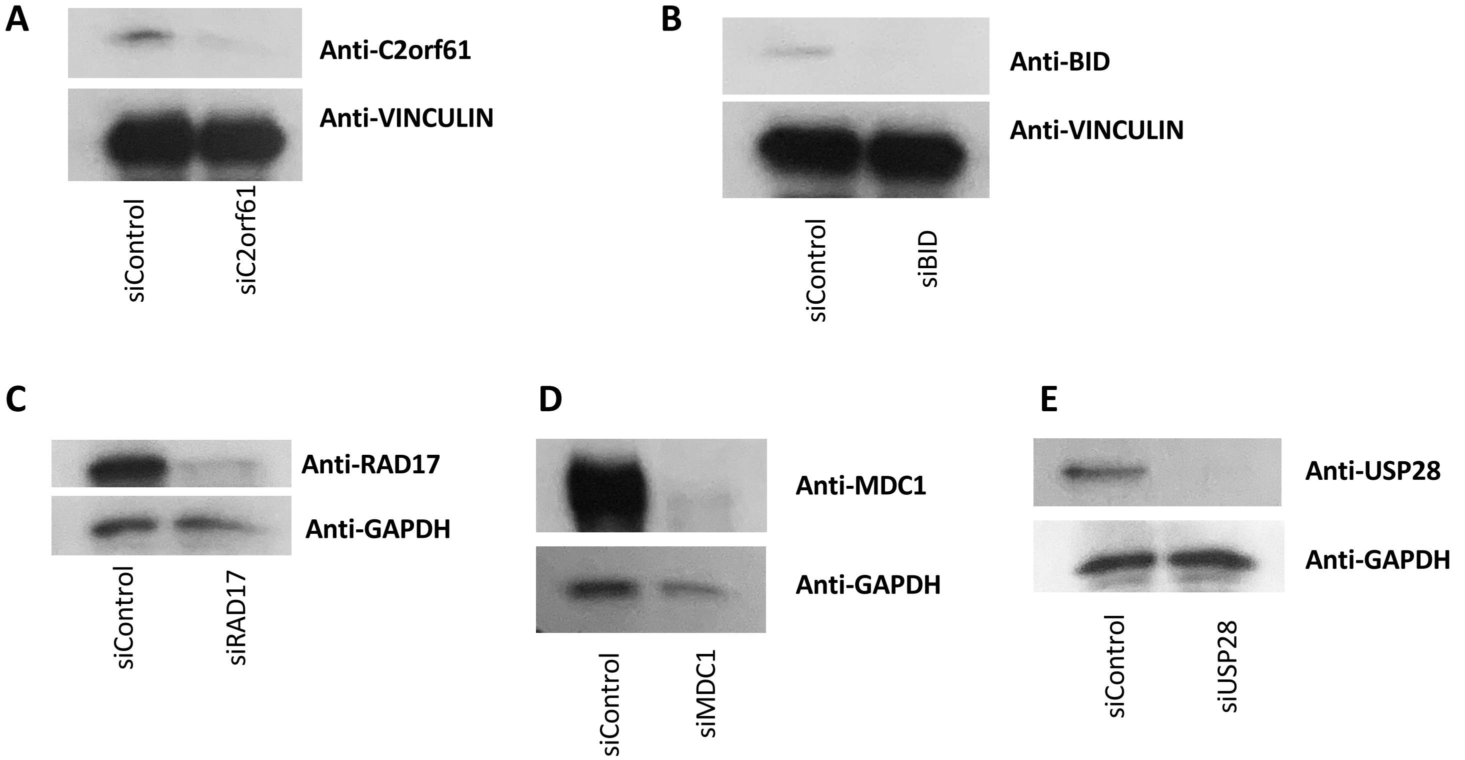

Supplement: Supplementary file 1 — Supplementary Material. [file 41389_2022_410_MOESM1_ESM.pdf]
